# Quantitative Measurement of Serum HBcrAg Can Be Used to Assess the Feasibility of Safe Discontinuation of Antiviral Therapy for Chronic Hepatitis B

**DOI:** 10.3390/v16040529

**Published:** 2024-03-29

**Authors:** Yong-Hong Wang, Hong Tang, En-Qiang Chen

**Affiliations:** Center of Infectious Diseases, West China Hospital, Sichuan University, Chengdu 610041, China; wangyongh@scu.edu.cn

**Keywords:** HBcrAg, hepatitis B virus, chronic hepatitis B, drug withdrawal

## Abstract

Hepatitis B virus (HBV) infection is a serious global health problem, and chronic HBV infection significantly increases the risk of liver fibrosis, cirrhosis, and even hepatocellular carcinoma in patients. Current first-line therapeutics such as nucleos(t)ide analogues and interferons are unable to completely clear cccDNA, so the vast majority of patients need to take long-term or even lifelong medication. However, long-term virological and biochemical responses can be achieved in some patients after drug withdrawal. Successfully screening these patients with drug withdrawal advantages is difficult. Hepatitis-B-core-related antigen (HBcrAg) is a new HBV serological marker that which can reflect the level and transcription activity of cccDNA in hepatocytes. Therefore, HBcrAg has potential value in guiding patients in drug withdrawal. This review summarizes previous reports on HBcrAg and evaluates the application value of HBcrAg in safe drug discontinuation.

## 1. Introduction

Hepatitis B virus (HBV) infection is a serious global health problem, and chronic HBV infection significantly increases the risk of liver fibrosis, cirrhosis, and even hepatocellular carcinoma (HCC) in patients [1]. The research report by Polaris Observatory Collaborators indicates that the global HBV prevalence in 2022 is estimated to be 3.2%, corresponding to 257.5 million HBsAg-positive individuals [2]. In China, the prevalence of HBsAg among the general population in 2021 was 3%, and the estimated chronic HBV infection was 43.3 million [3]. China accounts for a significant portion of the global burden of HBV infections and plays a key role in achieving the World Health Organization’s 2030 global hepatitis elimination target [3]. The persistent presence of covalently closed circular DNA (cccDNA) in hepatocytes is a key obstacle for a cure of chronic hepatitis B (CHB) [4]. Current first-line therapeutics such as nucleos(t)ide analogues (NAs) and interferons (IFN) are unable to completely clear cccDNA. Therefore, the goal of antiviral therapy for CHB is to maximize long-term inhibition of HBV replication, reduce liver inflammation and fibrosis, and reduce the occurrence of liver failure, cirrhosis decompensation, hepatocellular carcinoma (HCC), and other complications [5].

CHB treatment is a long-term process that can bring many adverse effects to patients, such as frequent medical visits that take up too much time, economic burden related to drug costs, and concerns about adverse drug reactions. Consequently, it is of great significance for CHB patients to be able to stop antiviral treatment safely. Long-term NAs treatment will gradually reduce the levels of cccDNA and HBsAg. However, the seroclearance rate of HBsAg is only 0~3%, and the recurrence rate after the withdrawal of NAs is high, so the vast majority of patients need to take long-term or even lifelong medication [6,7,8]. However, after long-term antiviral treatment, many CHB patients experience significant depletion of HBV cccDNA in their liver, and a considerable number of patients experience a significant decrease or even loss of serum HBsAg, indicating a significantly reduced risk of future liver cirrhosis and HCC. Therefore, more and more clinicians are pondering and discussing whether these CHB patients can terminate antiviral therapy.

## 2. Current Standards for Discontinuing Antiviral Therapy

Regarding the treatment duration of NAs, three major guidelines, including the European Association for the Study of Liver Disease (EASL), the Asian Pacific Association for the Study of Liver Disease (APASL), and the American Association for the Study of Liver Disease (AASLD), provide many recommendations [9,10,11]. For patients without cirrhosis, all three guidelines indicate that NAs can be stopped after at least 12 months of consolidation therapy when hepatitis B e antigen (HBeAg)-positive CHB patients achieve HBeAg seroconversion, with undetectable HBV DNA and persistently normal ALT. However, the three guidelines have different recommendations on the NAs cessation criteria for HBeAg-negative CHB patients. In HBeAg-negative patients without cirrhosis, the APASL proposes that NAs should be discontinued if one of the following conditions is met: (1) anti-HBs seroconversion or consolidation therapy for at least 12 months after HBsAg loss or (2) at least two years of antiviral treatment and three instances of undetectable HBV DNA, 6 months apart [10]. Treatment discontinuation may be considered in HBeAg-negative patients with HBsAg disappearance without cirrhosis, but the risks and benefits of discontinuation must be carefully evaluated. Discontinuation of treatment in patients with cirrhosis is not recommended [11]. Discontinuation of NAs may be considered in non-cirrhotic HBeAg-negative patients with long-term virologic suppression (≥3 years) after NAs therapy if close monitoring after NAs discontinuation is guaranteed [9]. Notably, although each guideline provides corresponding NAs discontinuation criteria, they all emphasize the need for close follow-up after discontinuation. The current safety discontinuation standards are only relatively safe, and there is still a risk of virological and clinical relapse. The concept of CHB cure, including complete cure and clinical cure, is mainly proposed to achieve safer drug withdrawal [12,13]. Complete cure means that serum HBsAg is undetectable; intrahepatic and serum HBV DNA is eliminated, including intracellular cccDNA and integrated HBV DNA; and serum hepatitis B core antibody (anti-HBc) remains positive, with or without the presence of anti-HBs [5]. Complete cure is the best indication for discontinuation, but it is difficult to achieve because of the persistence of cccDNA and the lack of specific targeted drugs for cccDNA. After completion of a defined course of treatment, clinical cure is characterized by persistent undetectability of serum HBsAg and HBV DNA, negative HBeAg with or without HBsAg seroconversion, persistence of residual cccDNA, remission of liver inflammation and improvement of liver histopathology, and a significant reduction in the incidence of end-stage liver disease [5]. Clinical cure is the ideal end point for discontinuation that can be achieved with existing antiviral drugs, but only a small percentage of CHB patients complete it [14,15].

## 3. Serum HBcrAg Can Reflect the Transcription Level of Intrahepatic HBV cccDNA

Given that current antiviral drugs cannot clear cccDNA, and HBsAg does not disappear in some discontinued patients, nearly half of the discontinued patients experience virological relapse regardless of pre-treatment HBeAg status [16]. Studies have shown that HBsAg loss after NAs treatment is related to good clinical outcomes and is persistent during long-term follow-up [7]. Unfortunately, the endpoint of HBsAg loss can only be achieved in <5% of NAs-treated patients, and achieving this standard is difficult [15]. The presence of cccDNA in hepatocytes is a major cause of virological relapse after discontinuing antiviral therapy [17]. Therefore, direct detection of intrahepatic cccDNA levels is the best method to predict virological relapse after drug withdrawal. However, detecting cccDNA requires liver biopsy, an invasive procedure that is difficult to promote in patients. In addition, due to sampling errors and lack of standardization of measurements, more studies are needed for the clinical application of cccDNA quantification [18,19]. Recently, some researchers have proposed that some HBV serological biomarkers can reflect the cccDNA level to some extent, such as HBsAg [20], HBeAg [21], HBV DNA [22], HBV RNA [23], hepatitis-B-core-related antigen (HBcrAg) [24], anti-HBc [25], etc. HBsAg can be translated from the mRNA produced by cccDNA and thus can partly reflect the level of cccDNA in hepatocytes. In one study, there was a correlation between serum HBsAg concentration and hepatic cccDNA levels: the highest in HBeAg-positive CHB patients and the lowest in patients with regressed hepatitis [26]. HBeAg seroconversion in a natural history of CHB is associated with a significant decrease in serum HBV DNA and a reduction in hepatic cccDNA content [27]. Therefore, HBeAg only reflects higher cccDNA levels in HBeAg-positive patients compared to HBeAg-negative patients. There is a good correlation between HBV DNA and cccDNA in untreated CHB patients [28]. However, when HBV DNA is undetectable, intrahepatic cccDNA persists in treated CHB patients. HBV RNA can reflect the intrahepatic transcription activity of cccDNA in untreated and NAs-treated CHB patients [29]. Similarly, serum HBV RNA was strongly correlated with intrahepatic cccDNA levels before and after 48-week IFN treatment [30]. Unfortunately, the detectability of HBV RNA was significantly reduced after NAs treatment and was inversely correlated with the duration of treatment [31]. Meanwhile, the molecular characteristics of serum HBV RNA have not been well defined, and there is no standardized method for detecting serum HBV RNA [32]. The anti-HBc antibody level of HBV cccDNA-positive patients was significantly higher than that of negative patients, and an anti-HBc IgG value higher than the 4.4 cut-off index (COI) was associated with positive cccDNA in the liver [33]. In a study of 44 patients with previous HBV infection, anti-HBC ≥ 8.9 S/CO was associated with cccDNA detectability [34]. Therefore, the detection of anti-HBc is mainly used for the evaluation of intrahepatic cccDNA in HBsAg-negative and anti-HBc-positive patients. HBcrAg is a new serological marker of HBV. Some studies have shown that the serum HBcrAg level can reflect the level and transcriptional activity of cccDNA in hepatocytes [35,36]. Hepatic cccDNA levels and cccDNA activities were lower in HBcrAg-negative (<3 Log U/mL) patients than in HBcrAg-positive patients [37]. In patients with different disease stages of CHB, the correlation between HBcrAg and intrahepatic cccDNA was stronger than HBsAg and HBV DNA [38]. Chen et al. found that HBcrAg, HBsAg, and HBV RNA were all correlated with cccDNA levels in HBeAg-positive patients, but only serum HBcrAg was correlated with cccDNA levels in HBeAg-negative patients [39]. Consequently, HBcrAg may be an excellent discontinuation indicator.

HBcrAg is a complex containing three HBV proteins, namely HBeAg, hepatitis B core antigen (HBcAg), and truncated HBcAg (p22cr), which share identical 149-amino-acid sequences [40]. In 2002, Kimura et al. first developed an enzyme immunoassay to detect HBcrAg and found that HBcrAg concentration has a similar effect as HBV DNA in reflecting HBV viral load [41]. The first-generation chemiluminescence enzyme immunoassay (CLEIA) HBcrAg test kit was used to detect serum HBcrAg, which can simultaneously measure denatured HBeAg, HBcAg, and p22cr. The measurement range of the HBcrAg assay kit is 3~7 log U/mL. HBcrAg below 3 log U/mL is recognized as 3 log U/mL. Samples with HBcrAg above 7 log U/mL are diluted and retested to detect the quantitative level of HBcrAg [42]. Notably, the relative contribution of each HBcrAg component in current commercial kit testing is unknown and may be influenced by factors such as viral genotype and HBeAg status, which may affect the accuracy and significance of HBcrAg as a biomarker [43]. Studies have shown that HBeAg is the main component of HBcrAg in HBeAg-positive patients (71.98 ± 10.24%), while HBcAg and p22cr only account for 17.64 ± 8.84% and 15.80 ± 9.13% of HBcrAg, respectively [44]. Undetectable HBcrAg (<3 log U/mL) is frequently encountered in HBeAg-negative patients, indicating the need for a more sensitive HBcrAg assay. Recently, a new highly sensitive assay (iTACT-HBcrAg) was developed to quantify HBcrAg in patients with CHB and was found to be almost ten times more sensitive than the traditional HBcrAg assay [45]. Quantitative analysis of iTACT-HBcrAg involves an automated pre-treatment process, and the assay is completed in approximately 30 min using a fully automated system. In 161 patients with negative HBeAg and persistent undetectable HBV DNA, HBcrAg can be detected in 97.5% of serum by iTACT-HBcrAg, among which 75.2% HBcrAg ≥ 2.8 log U/mL, and 22.4% HBcrAg are between 2.1–2.8 log U/mL, which cannot be detected by conventional HBcrAg detection methods [45]. Despite its increased sensitivity, HBcrAg cannot be detected by iTACT-HBcrAg in some CHB patients [45]. Therefore, more sensitive HBcrAg detection methods need to be further studied.

## 4. Evidence of Serum HBcrAg in Predicting Discontinuing Antiviral Therapy

When both HBsAg and HBV DNA are undetectable, HBcrAg can still be detected in some patients [46]. A study of 222 Chinese CHB patients (90 HBeAg-positive patients) treated with continuous ETV for 7 years showed that HBV DNA was undetectable in 98.7% of patients, while HBcrAg was still detectable in 68% of patients [47]. Similarly, a cohort study of 76 HBeAg-positive CHB patients treated with lamivudine (LAM) and adefovir (ADV) for 96 weeks showed that HBV DNA was undetectable in 48.7% (37/76) of patients, while HBcrAg was detectable in all patients [48]. This phenomenon may be due to the fact that NAs inhibit DNA synthesis by targeting HBV polymerase without affecting the formation of HBcrAg. In addition, HBcrAg may come from the transcription of cccDNA in the liver [35,36,49]. In 43 patients treated with NAs for a median of 126 months, HBV DNA was undetectable in 98%, while cccDNA was still detectable in 51% of patients [8]. Studies have confirmed that the decrease in HBcrAg has a good correlation with the change of cccDNA in the liver, while the correlation between HBV DNA and cccDNA is poor due to the inhibition of NAs [36,50]. Therefore, HBcrAg can better reflect the level of cccDNA than HBV DNA. Moreover, HBcrAg decreased gradually with the extension of NA treatment, but the decrease trend was lower than that of HBV DNA and HBsAg [46]. Wang et al. found that from baseline to year 8, serum HBcrAg levels in both HBeAg-negative and HBeAg-positive patients gradually decreased. After 8 years of NAs treatment, 21.3% of patients had serum HBcrAg < 3 log U/mL [51]. Clinical guidelines for the management of CHB in several countries recommend HBcrAg testing, first in Japan, then in Asia, and recently in Europe [52].

Many studies have confirmed that HBcrAg can predict virological and clinical relapse after NAs discontinuation. In a LAM discontinuation study with 34 patients, it was found that HBcrAg levels below 4.5 log U/mL may reduce the risk of HBV reactivation after discontinuation, corresponding to an area under the receiver-operating-characteristic curve (AUROC) of 0.764 [53]. However, a similar study showed that an HBcrAg level < 3.4 log U/mL at LAM discontinuation was the only independent predictor of no relapse after treatment [54]. Jung et al. showed that 26 (57.8%) HBeAg-positive and 37 (54.4%) HBeAg-negative patients had virologic relapse within one year after NAs discontinuation. End-of-treatment (EOT) HBcrAg level > 3.7 log IU/mL was a risk factor for predicting virologic relapse in HBeAg-negative patients [55]. The cumulative 4-year clinical relapse and virological relapse in patients with HBcrAg levels < 4 log U/mL were significantly lower than those with HBcrAg levels ≥ 4 log U/mL. In addition, HBcrAg level ≥ 4 log U/mL at EOT independently predicted clinical relapse with a hazard ratio (HR) of 5.696, and the AUROC value of clinical relapse predicted by HBcrAg was 0.621 [23]. In a systematic review study, the virological and clinical relapse in patients with detectable EOT HBcrAg ranged from 53.0 to 74.1% and 39.5 to 48.3%, respectively, compared with 14.0 to 44.1% and 7.3 to 13.9% in patients with undetectable HBcrAg. The cut-off values for EOT HBcrAg ranged from undetectable (<2 or 3 log10 IU/mL) to 4.5 log IU/mL [56]. Among patients with EOT HBsAg < 100 IU/mL, the 5-year virological and clinical relapse for baseline HBcrAg ≤ 4 and >4 log U/mL were 15.6%, 46.5% and 4.3%, 35.7%, respectively [57]. Similarly, among patients with EOT HBsAg < 150 IU/mL, the five-year virological relapse rates of patients with baseline HBcrAg level ≤ 4 and >4 log U/mL were 27.9% and 59.1%, respectively, and the corresponding clinical relapse rates were 18% and 48.1%, respectively [58]. Therefore, compared with the HBcrAg level at EOT, the baseline HBcrAg level may also play an important role in predicting the safety of drug withdrawal. Among patients with baseline HBcrAg levels < 4.7 and ≥4.7 log U/mL, virological relapse at 36 months was 55.1% and 82.4%, respectively, and clinical relapse was 39.4% and 72.6%, respectively [42]. Huang et al. proposed that a baseline HBcrAg of 4 log IU/mL was the optimal cutoff for predicting virological relapse. In contrast, HBcrAg at EOT did not significantly predict virologic or clinical relapse after ETV discontinuation [58]. Some studies suggest that EOT HBcrAg is not a significant predictor of virologic or clinical relapse after NAs discontinuation [21,58,59]. Therefore, there is still some controversy regarding the value of baseline and EOT HBcrAg in predicting NAs discontinuation. Overall, most studies support HBcrAg as a predictor of virologic and clinical relapse after NAs discontinuation.

In addition to the use of HBcrAg alone to predict relapse after NAs withdrawal, there are also many reports on the combination of HBcrAg with other virological indicators. In patients with HBsAg less than 100 IU/mL after treatment with entecavir or TDF, the investigators found that virological and clinical relapse rates within five years were low (<10%) in patients with EOT HBsAg levels lower than 40 IU/mL combined with baseline HBV DNA levels lower than 5 × 10^4^ IU/mL or baseline HBcrAg levels lower than 4 log U/mL [60]. An EOT HBsAg level of 20 IU/mL and a baseline HBcrAg level of 4 log U/mL were the best cut-off values for predicting 5-year virological and clinical relapse, corresponding to relapse rates of 6.5% and 0%, respectively [57]. In a similar study, an EOT HBsAg level of 100 IU/mL and a baseline HBcrAg 4.7 log U/mL were used to assess the risk of 3-year virological and clinical relapse, corresponding to relapse rates of 20.3% and 10.3%, respectively [42]. In addition, an EOT HBsAg of 150 IU/mL and a baseline HBcrAg of 4 log U/mL were found to be effective in predicting the risk of HBV recurrence in HBeAg-negative patients after entecavir discontinuation [58]. In a global research cohort, the results show that patients with low HBsAg (<100 IU/mL) and/or undetectable HBcrAg level (<2 log IU/mL), especially non-Asians or patients infected with HBV genotype C, seem to be the best candidates for discontinuation [61]. In the guidelines of the Japan Society of Hepatology on the treatment of HBV infection, it is pointed out that low serum HBsAg (<80 IU/mL) and HBcrAg (<3.0 log IU/mL) can predict a lower risk of relapse after NAs withdrawal [62]. However, some studies have suggested that the combination of HBsAg and HBcrAg does not demonstrate better predictive value for relapse after NAs withdrawal [63]. This phenomenon may be because HBsAg can be produced by cccDNA, and integrated HBV DNA and cannot reflect the level and transcriptional activity of cccDNA like HBcrAg, especially in HBeAg-negative patients [39,64]. With the extension of the NAs treatment course, cccDNA and HBsAg will gradually decrease. In addition, patients with low levels of HBsAg at the time of NAs withdrawal are more likely to achieve the disappearance of HBsAg [65,66], and the disappearance of HBsAg is one of the key components of functional cure. Therefore, although some HBsAg may be derived from integrated HBV DNA, low levels of HBsAg are positively correlated with hepatic cccDNA levels and predict the risk of discontinuation. In addition to the combination of HBsAg, the combination of HBcrAg and HBV RNA has also been reported in the prediction of relapse after NAs withdrawal. Kaewdech et al. found that HBcrAg and HBV RNA at EOT were independently associated with clinical relapse after discontinuation. Patients with undetectable HBcrAg (<3.0 log U/mL) and HBV RNA (<2.0 log copies/mL) at EOT did not experience clinical relapse during follow-up [63]. There was no clinical relapse in patients with negative HBV RNA and HBcrAg < 4 log U/mL, whereas 46.8% of patients with positive HBV RNA and HBcrAg ≥ 4 log U/mL experienced clinical relapse at 4 years of follow-up after discontinuation [23,67]. The AUROC values of HBsAg, HBcrAg, and HBV RNA for predicting virological relapse were 0.607, 0.686, and 0.648, respectively. Accordingly, as a single biomarker, serum HBcrAg is superior to HBV RNA and qHBsAg in predicting virological relapse [63]. Similarly, it has been found that serum HBcrAg correlates better with intrahepatic cccDNA levels than HBV RNA and HBsAg, regardless of HBeAg status [39].

Functional cure has many similarities with occult HBV infection (OBI) [68]. Anti-HBc can be the only detectable serological marker of OBI, persisting during both HBV infection and recovery [69]. However, only a few anti-HBc-positive individuals belong to OBI [70]. The diagnosis of OBI can be confirmed by detecting HBV DNA in the liver tissue or serum of anti-HBc-positive individuals [71]. For OBI patients, if NAs are used as prophylactic antiviral therapy, discontinuation can be attempted 6–12 months after completion of chemotherapy, targeted therapy, or immunosuppressive therapy [72]. Seto et al. found that serum HBcrAg positivity is an important risk factor for HBV reactivation in OBI patients receiving immunosuppressive therapy [73]. Most patients with HBsAg seroclearance (79%) had undetectable levels of HBcrAg, indicating a more quiescent state of HBV replication. In the remaining 21% of serum HBcrAg-positive patients, the median level of HBcrAg was 2.7 log U/mL [74]. Similarly, in another study, the median concentration of HBcrAg at discontinuation was 3.9 log IU/mL in patients with HBsAg seroclearance [75]. Interestingly, some scholars evaluated the effectiveness of iTACT-HBsAg and iTACT-HBcrAg detection in 96 CHB patients with HBsAg clearance and found that ten years after HBsAg clearance, HBsAg and HBcrAg could be detected in 20.4% and 64.5% of patients, respectively [40]. This may be due to the presence of HBV DNA in the form of cccDNA in the liver even after HBsAg seroclearance [76]. Although a functional cure is the ideal treatment endpoint, virological and clinical relapses may occur in some patients after discontinuation. Huang et al. found that the positive predictive value of HBcrAg < 4 log U/mL combined with HBsAb > 2 log IU/L for a durable functional cure was 100%, and the corresponding AUROC was 0.822 [77]. In other words, functionally cured CHB patients may still have a low level of HBV protein expression, and HBcrAg may be used for long-term monitoring of these patients [75].

## 5. Conclusions

The persistent presence of cccDNA in hepatocytes is a key obstacle to curing CHB. However, current first-line therapeutics are unable to completely clear cccDNA. Long-term NAs treatment will gradually reduce the levels of cccDNA and HBsAg, but the recurrence rate after NAs withdrawal is high, so the vast majority of patients need to take long-term or even lifelong medication. CHB treatment is a long-term process that can bring many adverse effects to patients, such as frequent medical visits that take up too much time, economic burden related to drug costs, and concerns about adverse drug reactions. Consequently, it is of great significance for CHB patients to stop antiviral treatment safely. The current safety discontinuation standards recommended by the three major liver disease guidelines are only relatively safe, and there is still a risk of virological and clinical relapse. HBcrAg is a new serological marker of HBV. Some studies have shown that serum HBcrAg levels can reflect the level and transcriptional activity of cccDNA in hepatocytes. Accordingly, HBcrAg may be an excellent discontinuation indicator.

HBcrAg is a complex containing three HBV proteins that can be detected by enzyme immunoassay. Recently, iTACT-HBcrAg was developed to quantify HBcrAg in patients with CHB and was found to be almost ten times more sensitive than the traditional HBcrAg assay. The iTACT-HBcrAg assay can detect low concentrations of HBcrAg (2.1 Log U/mL). The advancement of this detection method will greatly promote the application of HBcrAg. The ability of HBcrAg to predict virologic and clinical relapse after NAs discontinuation has been confirmed by many studies, but the determination of the optimal predictive cut-off value of HBcrAg is still controversial. In addition, there are different opinions about the baseline and EOT values of HBcrAg in predicting relapse after NAs discontinuation. Some scholars recommend using baseline HBcrAg for prediction, while others recommend EOT HBcrAg for prediction, and even some studies support combining baseline and EOT for prediction [42]. However, it is clear that the lower the level of HBcrAg, the lower the risk of relapse after withdrawal, regardless of baseline or EOT. In addition to the use of HBcrAg alone to predict relapse after NAs withdrawal, there are also many reports on the combination of HBcrAg with other virological indicators (Table 1). The most commonly used predictive biomarkers in combination with HBcrAg are HBV RNA and HBsAg. Moreover, the Japanese Society of Hepatology guidelines recommend the combination of HBcrAg and HBsAg. The combination of HBcrAg and other viral markers to predict drug withdrawal has a higher safety and deserves further study. It is noteworthy that the relapse of drug withdrawal is influenced by many factors, such as virus subtype, host, and drug type. Especially for drugs, both virologic and clinical relapse after TDF discontinuation were found to be significantly higher than ETV [78,79]. In addition to predicting relapse after NAs withdrawal, HBcrAg has been reported to predict HBeAg seroconversion, HCC occurrence or recurrence, HBV reactivation, HBsAg loss, and antiviral treatment response [24]. Interestingly, although NAs withdrawal may lead to virological and clinical relapse, it increases the chance of HBsAg loss. Patients with HBsAg loss can obtain a functional cure, which is the ideal endpoint of HBV treatment. Some scholars have found that EOT HBsAg level < 1000 IU/mL is a key predictor of HBsAg loss [66]. Similarly, patients with HBsAg < 100 IU/mL and HBV DNA < 100 IU/mL at NAs discontinuation had a lower risk of relapse and better HBsAg loss [80]. In addition, low HBsAg levels (<100 IU/mL) and undetectable HBcrAg levels at NA discontinuation were associated with higher HBsAg loss [61]. Whether HBcrAg is superior to HBsAg in predicting relapse and HBsAg loss after NAs withdrawal remains to be studied. However, the combination of HBcrAg and HBsAg should have a higher predictive value.

**Figure 1:**
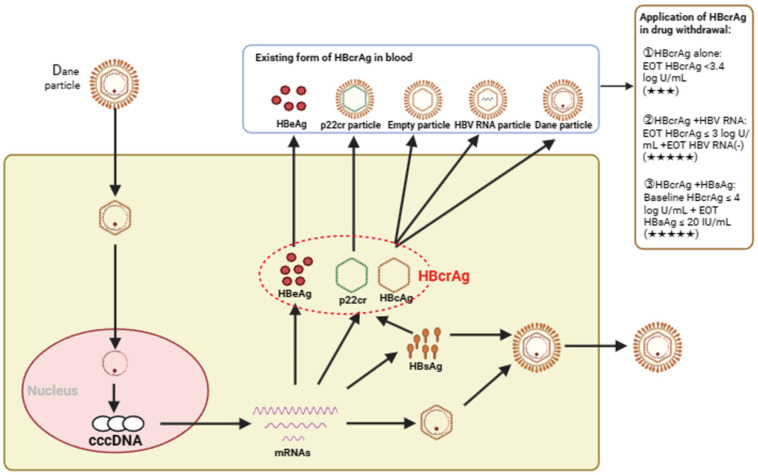
The generation, existence form, and application of HBcrAg in drug withdrawal. HBcrAg is a complex containing three HBV proteins: HBeAg, HBcAg, and p22cr. HBcrAg was mainly derived from HBeAg, p22cr particles, empty capsid, HBV RNA particles, and Dane particles [31]. cccDNA, covalently closed circular DNA; HBcrAg, hepatitis-B-core-related antigen; HBeAg, hepatitis B e antigen; HBcAg, hepatitis B core antigen; HBsAg, hepatitis B s antigen; p22cr, truncated HBcAg; EOT, end of treatment; ★★★, moderate recommendation; ★★★★★, strong recommendation.

In conclusion, HBcrAg has great potential for application as a new serum marker for HBV (Figure 1). Although the cut-off value for HBcrAg to predict relapse after discontinuation has not been uniformly established, lower levels of HBcrAg often indicate greater discontinuation safety. After NAs treatment, an advantageous population can achieve clinical cure by IFN treatment, and these patients have a strong need for discontinuation. However, there are few reports about HBcrAg predicting relapse after clinical cure, especially in patients with clinical cure after IFN therapy. Therefore, more research data are needed to support the role of HBcrAg in the safety of discontinuation in clinically cured patients. In China, most hospitals do not include HBcrAg in routine testing, and medical insurance cannot be reimbursed, which leads to very few patients being tested for HBcrAg. Therefore, there is a lack of real-world data on HBcrAg testing in discontinued patients. Compared with traditional HBV serological indicators, HBcrAg has more advantages in predicting the risk of relapse after drug withdrawal, and with the popularization of high-sensitivity detection kits and the reduction of prices, the advantages of HBcrAg will become more significant. It is worth noting that the safety of drug withdrawal is relative before cccDNA is completely clear. Although new indicators such as HBcrAg can predict the safety of drug withdrawal, it is very necessary to follow up after drug withdrawal.

## Figures and Tables

**Table 1:** HBcrAg alone or in combination with other indicators predicted the risk of relapse after discontinuation.

Biomarkers	No. of Subjects	Drugs	Drug Withdrawal Time	VR(%)	CR(%)	Cut-Off Value	Ref.
HBcrAg	34	LAM	12 months	-	0	EOT HBcrAg < 4.5 log U/mL	[53]
	22	LAM	28 months	-	0	EOT HBcrAg < 3.4 log U/mL	[54]
	68	LAM, ETV	12 months	0	-	EOT HBcrAg ≤ 3.7 log U/mL	[55]
HBcrAg + HBV RNA	127	LDT	48 months	-	0	EOT HBcrAg < 4 log U/mL + EOT HBV RNA(-)	[23]
	92	LAM, LDT, ADV, ETV, TDF	12 months	-	0	EOT HBcrAg ≤ 3 log U/mL + EOT HBV RNA(-)	[63]
HBcrAg + HBsAg	31	ETV, TDF	60 months	6.5	0	Baseline HBcrAg ≤ 4 log U/mL + EOT HBsAg ≤ 20 IU/mL	[57]
	84	ETV	60 months	27.9	18	Baseline HBcrAg ≤ 4 log U/mL + EOT HBsAg < 150 IU/mL	[58]
	53	TDF	36 months	20.3	10.3	Baseline HBcrAg < 4.7 log U/mL + EOT HBsAg < 100 IU/mL	[42]
	36	ETV, TDF	60 months	5.9	2.8	Baseline HBcrAg < 4log U/mL + EOT HBsAg < 40 IU/mL	[60]

Abbreviations: HBcrAg, hepatitis-B-core-related antigen; LAM, lamivudine; LDT, telbivudine; ADV, adefovir; ETV, entecavir; TDF; tenofovir disoproxil fumarate; VR, virological relapse; CR, clinical relapse; EOT, end of treatment.

## Data Availability

No new data were created.
